# Method of Fatigue-Life Prediction for an Asphalt Mixture Based on the Plateau Value of Permanent Deformation Ratio

**DOI:** 10.3390/ma11050722

**Published:** 2018-05-03

**Authors:** Yazhen Sun, Chenze Fang, Jinchang Wang, Xuezhong Yuan, Dong Fan

**Affiliations:** 1 School of Transportation Engineering, Shenyang Jianzhu University, Shenyang 110168, China; syz16888@126.com (Y.S.); fangchenze@126.com (C.F.); 2 Institute of Transportation Engineering, Zhejiang University, Hangzhou 310058, China; wjc501@zju.edu.cn; 3 School of Science, Shenyang Jianzhu University, Shenyang 110168, China; xiaodaxia1122@126.com

**Keywords:** road engineering, fatigue life, three-point bending fatigue test, asphalt mixture, plateau value of permanent deformation ratio, damage evolution, fatigue equation

## Abstract

Laboratory predictions for the fatigue life of an asphalt mixture under cyclic loading based on the plateau value (PV) of the permanent deformation ratio (PDR) were carried out by three-point bending fatigue tests. The influence of test conditions on the recovery ratio of elastic deformation (RRED), the permanent deformation (PD) and PDR, and the trends of RRED, PD, and PDR were studied. The damage variable was defined by using PDR, and the relation of the fatigue life to PDR was determined by analyzing the damage evolution process. The fatigue equation was established based on the PV of PDR and the fatigue life was predicted by analyzing the relation of the fatigue life to the PV. The results show that the RRED decreases with the increase of the number of loading cycles, and the elastic recovery ability of the asphalt mixture gradually decreases. The two mathematical models proposed are based on the change laws of the RRED, and the PD can well describe the change laws. The RRED or the PD cannot well predict the fatigue life because they do not change monotonously with the fatigue life, and one part of the deformation causes the damage and the other part causes the viscoelastic deformation. The fatigue life decreases with the increase of the PDR. The average PDR in the second stage is taken as the PV, and the fatigue life decreases in a power law with the increase of the PV. The average relative error of the fatigue life predicted by the fatigue equation to the test fatigue life is 5.77%. The fatigue equation based on PV can well predict the fatigue life.

## 1. Introduction

In recent years, the road construction industry has made great progress with the development of technologies, but the qualities of existing roads are mixed. The asphalt concrete pavement, as the surface course of the road structure, is subjected to repeated actions of vehicle loads and the environmental influence of seasonal changes. Under cyclic loading, the stresses and strains in the material change continuously, which results in the reduction of the strength. Pavement under cyclic loading is prone to fatigue failure. The fatigue life of pavement material has become the focus of research for an increasing number of researchers [1,2,3,4,5,6,7,8]. Three approaches are usually used to study fatigue life: the phenomenological approach, the fracture-mechanics-based approach, and the energy (damage) approach [9,10,11,12,13,14,15].

The fracture mechanics approach was used to study fatigue by monitoring crack development in its early stages. Researchers carried out many studies on the fatigue properties of asphalt concrete with rubber grains based on fracture mechanics [16,17]. Principles of fracture mechanics were applied to data obtained by monitoring the size and the length of the crack opening to discover the stress intensity factors at the crack tip [18,19]. The Paris equation can describe the relation of the stress intensity factors to the crack propagation and was used to describe the growth process of fatigue cracks and predict the fatigue life of the asphalt mixture [20,21,22]. Limitations of this approach include the need for a large amount of experimental data, considering only the crack propagation, and the stress intensity factor *K*_I_ being not a material constant at higher temperatures [23,24]. Also, fracture mechanics cannot accurately describe the viscoelastic plastic mechanical properties of the asphalt mixture.

Researchers carried out many studies on the fatigue properties of bitumen and the asphalt mixture based on the dissipated energy concept. These studies assumed that fatigue life depends on the accumulation of dissipated energy in each loading cycle. The fatigue equation established based on this assumption was used to predict fatigue life [25,26,27,28]. Later studies demonstrated that the damage was related to the rate of change in dissipated energy from one loading cycle to the next and only part of the dissipated energy can cause damage to the material. Therefore, it is inaccurate to use the total dissipated energy to predict the fatigue life [29,30,31].

The phenomenological approach provides an important idea for the early study of fatigue properties. This approach assumes that the stress or the strain in the asphalt layer is related to the number of load repetitions to failure. Some fatigue equations established based on this assumption were used to predict fatigue life [32,33]. There was a large discreteness in the fatigue life predicted by these equations. Also, the phenomenological approach cannot reveal the mechanism of damage evolution [34,35,36,37].

The fatigue equations in the above studies, established from different perspectives, were used to predict the fatigue life of the asphalt mixture. These equations, established after processing a large amount of experimental data, were complex and inconvenient to use. Permanent deformation (PD) is accumulated during the damage evolution process of the asphalt mixture, and since the PD is easy to obtain and deal with, establishing the fatigue equation based on the analysis of the PD has important implications for researchers in predicting the fatigue life of the asphalt mixture.

To address the shortcomings of the above studies, understand the change law of deformation of the asphalt mixture under cyclic loading, and predict the fatigue life from the perspective of deformation, the AC-13I asphalt mixture was used under different experimental conditions to carry out the three-point bending fatigue tests in this paper. By analyzing the change laws of the recovery ratio of elastic deformation (RRED), the conclusions drawn from this are that the elastic recovery ability of the asphalt mixture under cyclic loading gradually decreases, and two mathematical models describing the change laws of the RRED and the PD are proposed. By analyzing the influence of experimental factors on the RRED and the PD, the conclusions also drawn are that the RRED or the PD does not change monotonously with fatigue life, and the RRED or the PD cannot be directly used to predict the fatigue life of the asphalt mixture under cyclic loading. The PD of the asphalt mixture does not change monotonously with fatigue life, indicating that only part of the PD causes the damage of the asphalt mixture, and that the other part causes the viscoelastic deformation. By analyzing the relationship between the damage evolution speed and the value of the permanent deformation ratio (PDR), it was found that the faster the damage evolution speed of the asphalt mixture under cyclic loading was, the earlier the damage was close to the threshold of failure, and the fatigue life of the asphalt mixture under cyclic loading decreased with the increase of the damage evolution speed. It was also found that the power function could be used to describe the mathematical relationship between fatigue life and the plateau value of the PDR of the asphalt mixture. The fatigue equation was established based on the power-function relationship between the plateau value of the PDR and the fatigue life. The predicted fatigue life of the asphalt mixture under different test conditions was calculated by the fatigue equation and compared with the test fatigue life. The relative error between the fatigue life predicted by the fatigue equation and the test fatigue life was small, which indicates that the proposed equation can be used to accurately predict the fatigue life of an asphalt mixture.

## 2. Materials and Experimental Procedures

### 2.1. Test Materials

A 70-penetration bitumen was used as asphalt binder for preparations of the specimens, with its specifications listed in Table 1. Limestone was used as the aggregate. The ratio of binder to aggregate is 8.1% by weight. The continuous aggregate gradation, having the nominal maximum size of 16 mm, is listed in Table 2.

### 2.2. Preparation of Specimen

**Figure 1:**
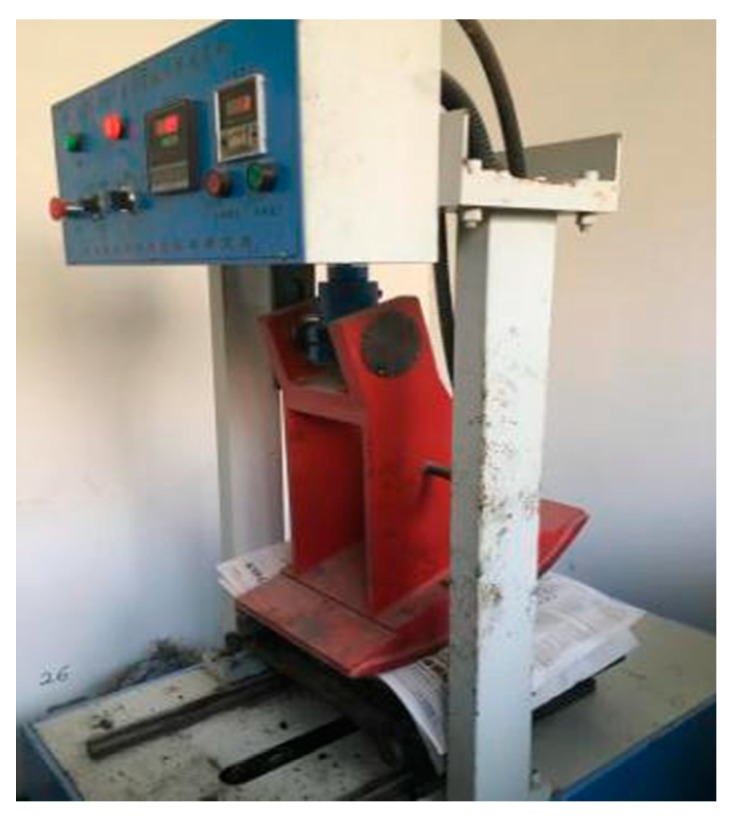
Rut board forming machine.

**Figure 2:**
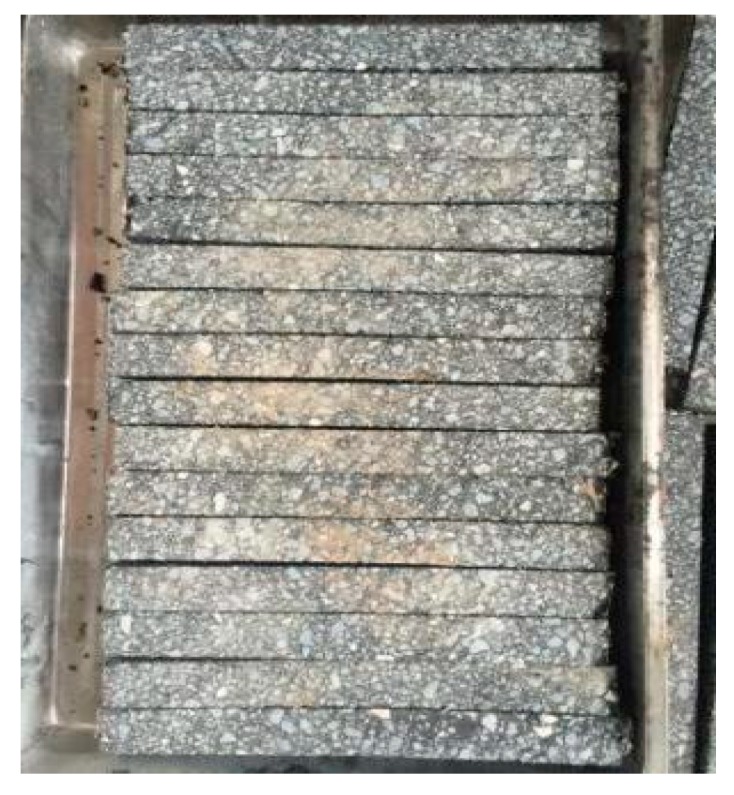
Specimen beams.

A rut board of 400 mm × 400 mm × 70 mm was made by a hydraulic sample forming machine [38], as shown in Figure 1. By cutting the rut board, specimen beams of 250 mm × 30 mm × 35 mm were obtained, as shown in Figure 2. The average density of the beam specimens is 2.445 g/cm^3^.

### 2.3. Test Conditions and Methods

**Figure 3:**
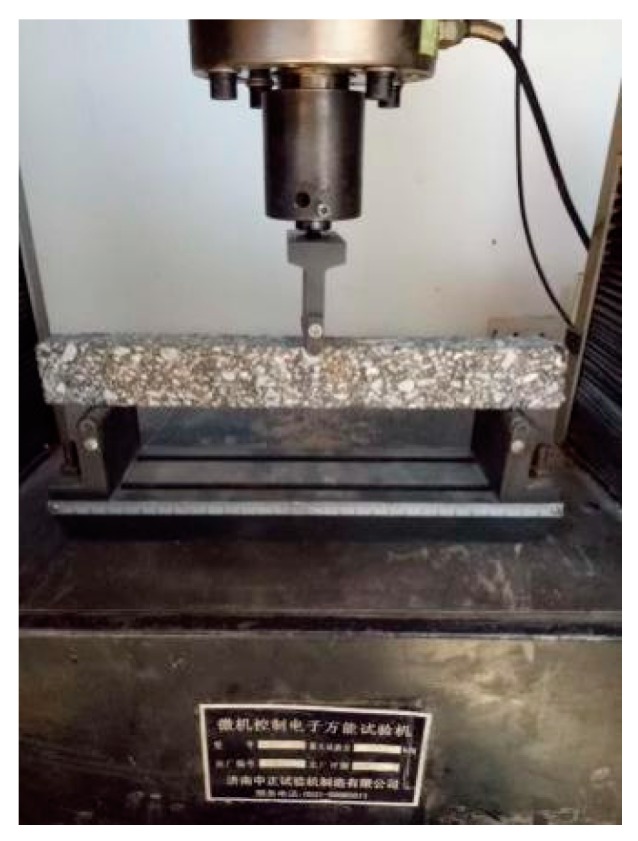
Fatigue test.

The fatigue test is shown in Figure 3. The flexural tensile strengths of specimens under different test conditions were measured. To better analyze the influence of experimental factors on test results, three groups of contrast tests were arranged:

Group 1. For this group, the temperature was 25 °C, the loading rate was 10 mm/min, and the stress-strength ratios (SSR) were 0.6, 0.7, and 0.8 respectively.
Group 2. The temperatures was 5 °C, 15 °C, and 25 °C, respectively, the loading rate was 10 mm/min, and the SSR was 0.6.
Group 3. The temperature was 25 °C, the loading rates were 10 mm/min and 20 mm/min, respectively, and the SSR was 0.6.

### 2.4. Test Results 

The fatigue tests were carried out according to test scheme and the fatigue lives are listed in Table 3. It can be seen that fatigue life decreases with the increase of the stress-strength ratio (SSR) and the loading rate and increases with the increase of the temperature.

## 3. Analysis of Recovery Ratio of Elastic Deformation

**Figure 4:**
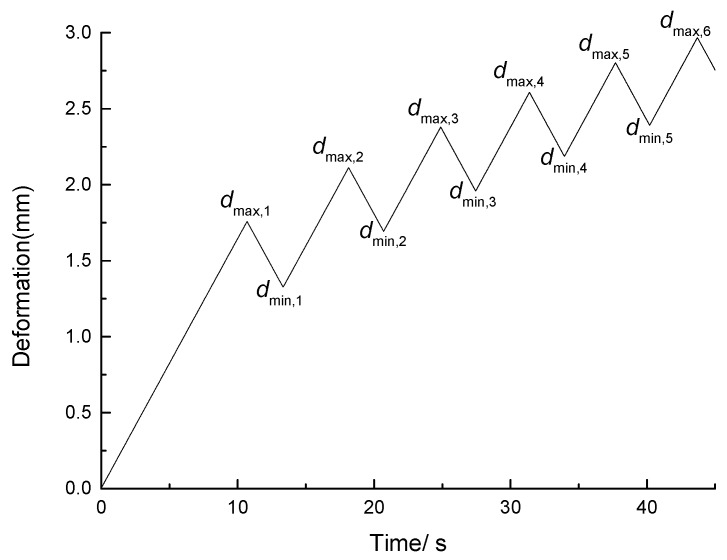
Deformation time.

The deformation time curve for the three-point bending fatigue test is shown in Figure 4. As shown in the figure, during each loading cycle, the deformation of the asphalt mixture increases linearly to the peak value at the set loading rate in the loading stage and decreases linearly to the point where the load is zero in the unloading stage. In this fashion, the asphalt mixture specimen is subjected to cyclic loading until it is broken. The elastic recovery ability of the asphalt mixture under cyclic loading gradually decreases and the RRED represents the elastic recovery ability of the asphalt mixture [31]. The RRED is given as

(1) $$\mathrm{RRED}=\frac{d_{\max,N}-d_{\min,N}}{d_{\max,N}}$$

where *d*_max__,*N*_ is the peak deformation of loading cycle *N* and *d*_min__,*N*_ is the minimum deformation of loading cycle *N*.

### 3.1. Construction of RRED Mathematical Model

**Figure 5:**
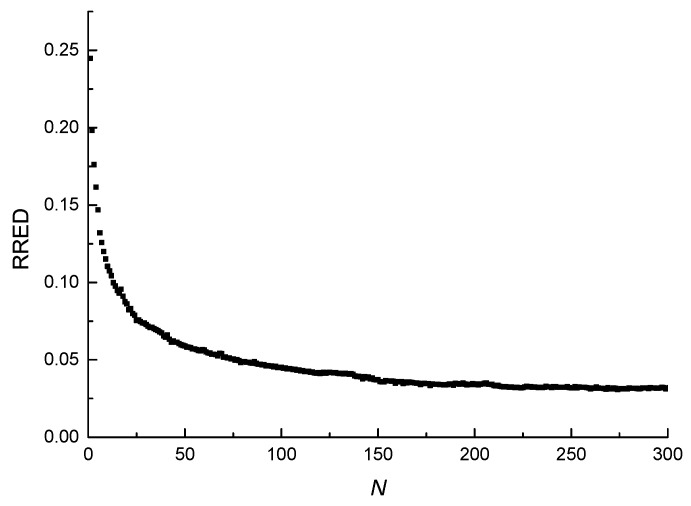
RRED-*N* curve.

The curve of the RRED vs. the number of loading cycles (RRED-*N* curve) of the three-point bending fatigue test calculated by Equation (1) is shown in Figure 5. As shown in the figure, the RRED decreases with the increase of the number of loading cycles, and the process can be divided into two stages. In the first stage, the RRED lasts a short time but decreases rapidly, and at the end of this stage, it drops to a small value. In the second stage, the RRED lasts a long time and decreases slowly. The RRED of the asphalt mixture decreases with the increase of the number of loading cycles, which indicates that both the elastic recovery ability and the integrity of the asphalt mixture decrease [39]. The RRED mathematical model was proposed to accurately describe the change law of the elastic recovery ability and the integrity of the asphalt mixture under cyclic loading. The RRED mathematical model is defined as

RRED = *aN^b^* (2)

where *a* and *b* are fitting parameters and *N* is the number of loading cycles.

**Figure 6:**
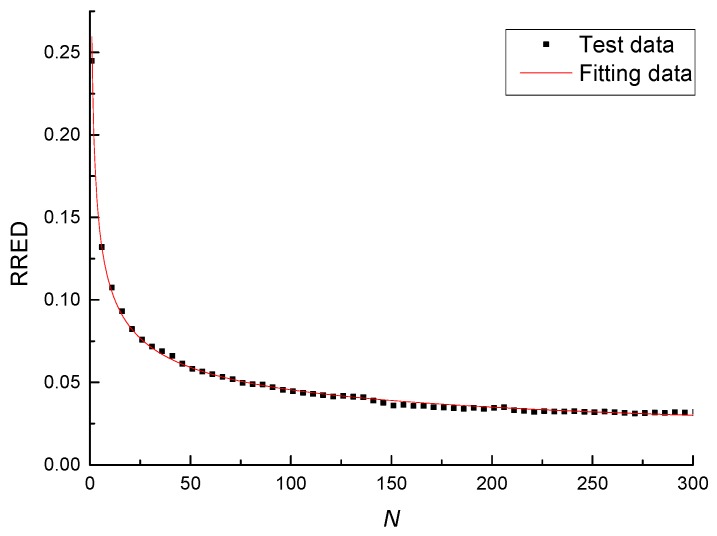
Test data and fitting data.

The proposed RRED mathematical model given by Equation (2) was used to fit the test data and the correlation coefficient is greater than 0.99, which indicates that the proposed model describes very well the elastic recovery ability and the integrity of the asphalt mixture under cyclic loading. The fitting results at the temperature of 25 °C, the stress-strength ratio of 0.6, and the loading rate of 10 mm/min are listed in Table 4, and the fitting data and test data are shown in Figure 6.

### 3.2. Influence of Experimental Factors on RRED

**Figure 7:**
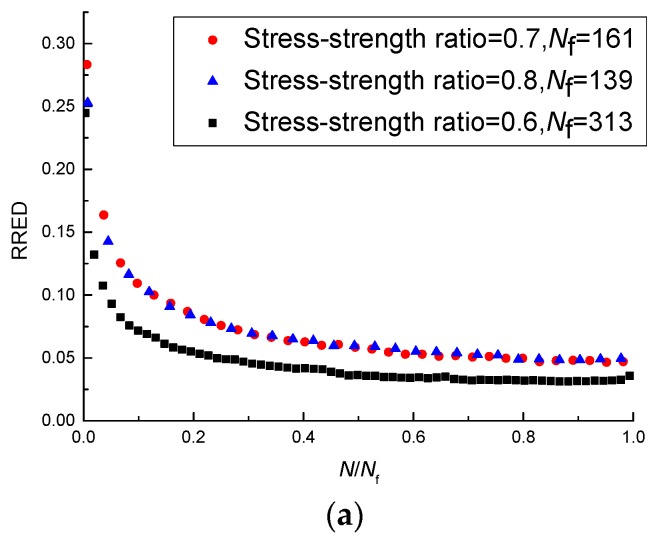

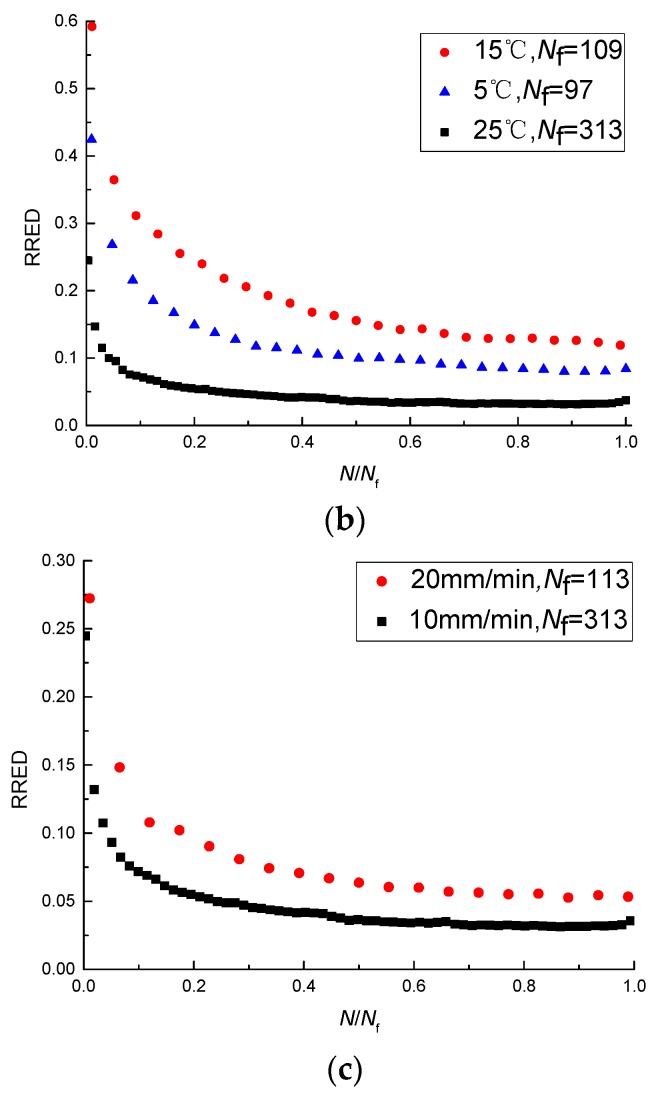
RREDs under different test conditions: (**a**) RREDs at different stress-strength ratios; (**b**) RREDs at different temperatures; (**c**) RREDs at different loading rates.

The results in different test conditions are shown in Figure 7. As shown in Figure 7a, the RRED calculated by Equation (1) at the stress-strength ratio of 0.7 is very close to that at the stress-strength ratio of 0.8, and they are greater than that at the stress-strength ratio of 0.6. However, the sequence of the magnitude of the fatigue life at different stress-strength ratios corresponds to the stress-strength ratios of 0.6, 0.7, and 0.8, respectively. As shown in Figure 7b, the RRED is influenced by the temperature, and the sequence of the magnitude of the recovery ratio at different temperatures corresponds to the temperatures of 15 °C, 5 °C, and 25 °C, respectively. However, the sequence of fatigue life corresponds to the temperatures of 25 °C, 15 °C, and 5 °C, respectively. As shown in Figure 7c, the RRED at the loading rate of 20 mm/min is greater than that at the loading rate of 10 mm/min, but the fatigue life at 10 mm/min is greater.

The above analyses indicate that the RRED does not change monotonously with the stress-strength ratio, the temperature, and the loading rate. It can be seen from Table 3 that there is a negative correlation between the fatigue life and the SSR and loading rate, and a positive correlation between the fatigue life and the temperature. So, the RRED does not change monotonously with the fatigue life and the RRED cannot well predict the fatigue life of the asphalt mixture.

## 4. Analysis of PD

### 4.1. Construction of PD Mathematical Model

The asphalt mixture, a viscoelastic plastic material subjected to cyclic loading, undergoes three different types of deformation: elastic deformation, viscous-flow deformation, and delayed deformation. After the unloading stage of each cycle, the elastic deformation of the asphalt mixture can fully recover, but the viscous-flow deformation of the asphalt mixture cannot recover. One part of the delayed deformation can recover, but the other part cannot recover [32]. The PD of the asphalt mixture under cyclic loading is composed of all the viscous-flow deformation and the delayed deformation that cannot recover. The fatigue failure of the beam specimen is mainly caused by the tensile stress at the bottom of the specimen, because the ultimate tensile stress of the asphalt mixture is far less than its ultimate compressive stress. When a stress produced by a cyclic loading is below the ultimate stress, the fatigue failure of the beam specimen is due to the stress concentration at the bottom, and the fatigue damage is mainly manifested by fatigue cracks. The growth process of a fatigue crack consists of two stages: the crack initiation stage and the crack propagation stage. Damage gradually accumulates with the increase of the number of loading cycles. Fatigue cracks occur at the bottom of the beam specimen when the damage is accumulated to a certain extent. Fatigue cracks expand due to the increase of the number of loading cycles. The cumulative result of the expansion is the fracture of the beam specimen [40]. The whole process of the damage evolution of the asphalt mixture is accompanied by the accumulation of the PD, and the accumulation of the PD will further the evolution of damage. Thus, the accumulation of the PD is related to the damage evolution and fatigue life of the asphalt mixture.

**Figure 8:**
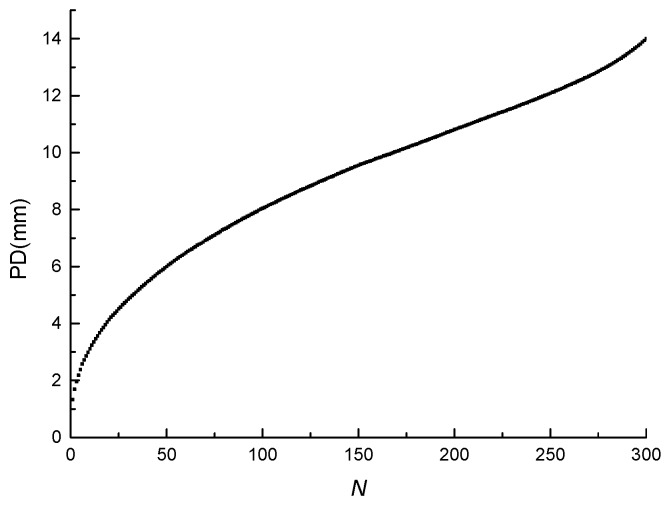
PD-*N* curve.

The change of the PD of the asphalt mixture under cyclic loading (vs. the number of loading cycles) is a monotonous accumulation process. The permanent deformation and the number of loading cycles (PD-*N*) curve of the three-point bending fatigue test is shown in Figure 8. As shown in the figure, the PD increases with the increase in the number of loading cycles, which can be divided into three stages. The first stage lasts a short time and the PD increases rapidly, but the growth rate gradually decreases. At the end of the first stage, the PD reaches a large value. The second stage lasts a long time and the PD increases stably. The third stage lasts a short time, but the PD increases rapidly and the growth rate gradually increases.

In order to accurately describe the change law of the PD of the asphalt mixture under cyclic loading (vs. the number of loading cycles), a PD mathematical model was proposed:

PD = *cN^d^* (3)

where *c* and *d* are fitting parameters and *N* is the number of loading cycles.

The model proposed was used to fit the experimental data of the PD and the correlation coefficient is greater than 0.98, which indicates that the proposed model can accurately describe the change law of the PD of the asphalt mixture under cyclic loading (vs. the number of loading cycles). The fitting results of the PD mathematical model are listed in Table 5.

### 4.2. Influence of Experimental Factors on PD

**Figure 9:**
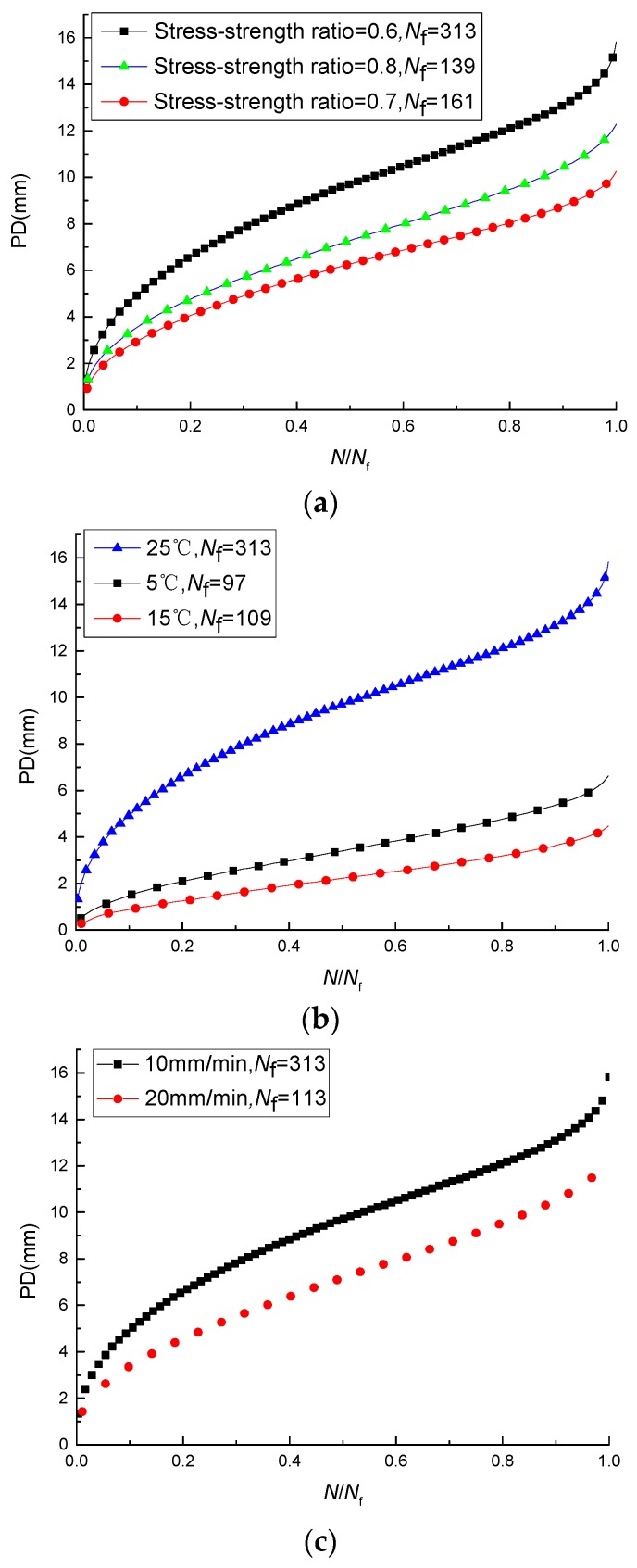
PDs under different test conditions: (**a**) PDs at different stress-strength ratios; (**b**) PDs at different temperatures; (**c**) PDs at different loading rates.

The results under different test conditions are shown in Figure 9. It can be seen from Figure 9a that the PD is obviously influenced by the stress-strength ratio. The sequence of the magnitude of the PD at different stress-strength ratios corresponds to the stress-strength ratios of 0.6, 0.8, and 0.7, respectively. However, the sequence of the magnitude of the fatigue life at different stress-strength ratios corresponds to the stress-strength ratios of 0.6, 0.7, and 0.8, respectively. As shown in Figure 9b, the PD is obviously influenced by the temperature. The sequence of the magnitude of the PD at different temperatures corresponds to the temperatures of 25 °C, 5 °C, and 15 °C, respectively. However, the sequence of the fatigue life at different temperatures corresponds to the temperatures 25 °C, 15 °C, and 5 °C, respectively. As shown in Figure 9c, the PD is obviously influenced by the loading rate. The PD at the loading rate of 10 mm/min is greater than that at 20 mm/min, and the fatigue life at the loading rate of 10 mm/min is also greater. It is evident that the PD of the asphalt mixture does not change monotonously with the fatigue life. Therefore, the PD of the asphalt mixture cannot well predict the fatigue life.

The process of damage evolution is accompanied by the accumulation of PD, and the accumulation of PD will further the evolution of damage. Therefore, the accumulation of the PD is related to the damage evolution speed. The faster the damage evolution speed, the sooner the damage is close to the threshold of the failure of the asphalt mixture, and the shorter the fatigue life is. Therefore, the fatigue life of the asphalt mixture is related to the PD. The PD of the asphalt mixtures does not change monotonously with the fatigue life, which indicates that all of the PD is not used to damage the asphalt mixture. One part of the PD causes the damage to the asphalt mixture under cyclic loading, and the other part causes the viscoelastic deformation of the asphalt mixture [41].

## 5. Analysis of PDR

The PD is related to damage evolution and fatigue life, but the PD cannot be directly used to predict the fatigue life of the asphalt mixture. To reveal the relationship between the PD and the fatigue life, the definition of PDR is proposed in this paper as

(4) $$\mathrm{PDR}=\frac{\left|{\mathrm{PD}}_{N+1}-{\mathrm{PD}}_{N}\right|}{{\mathrm{PD}}_{N}}$$

where PD*_N_*_+1_ and PD*_N_* are the PDs of the loading cycle (*N*+1) and the loading cycle *N*. Under certain conditions, the PD of the viscoelastic deformation part of each loading cycle is a fixed value [41]. The PDR reflects the proportion of the PD that produces the viscoelastic deformation to the total PD of each loading cycle. The value of PDR is only related to the damage deformation.

### 5.1. Influence of Experimental Factors on PDR

**Figure 10:**
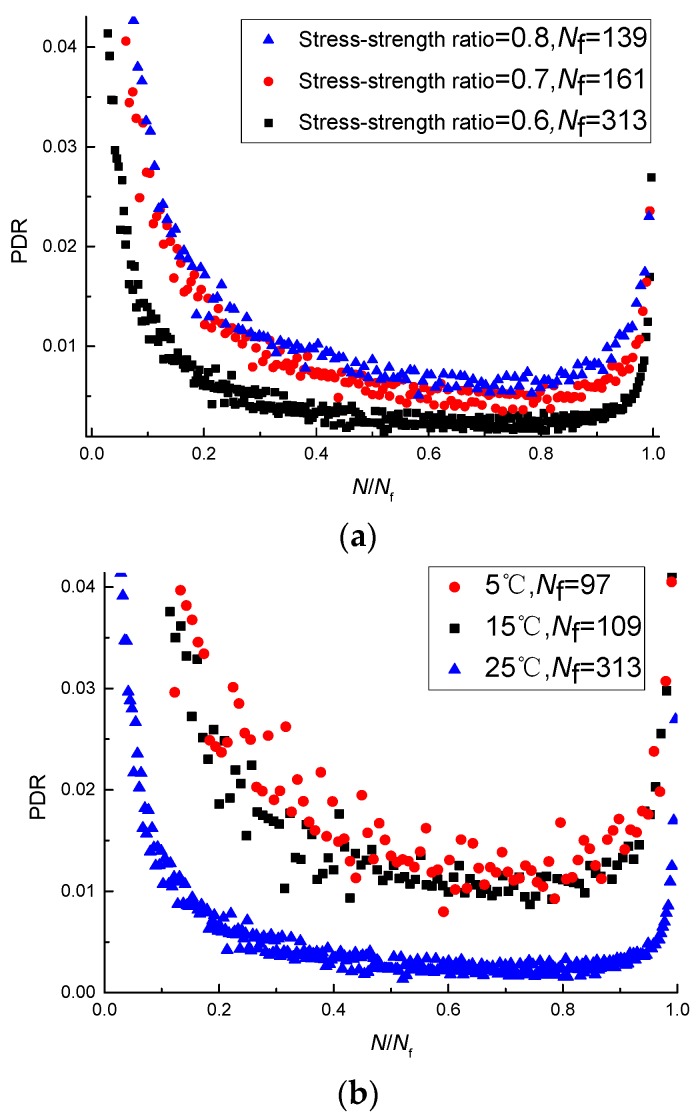

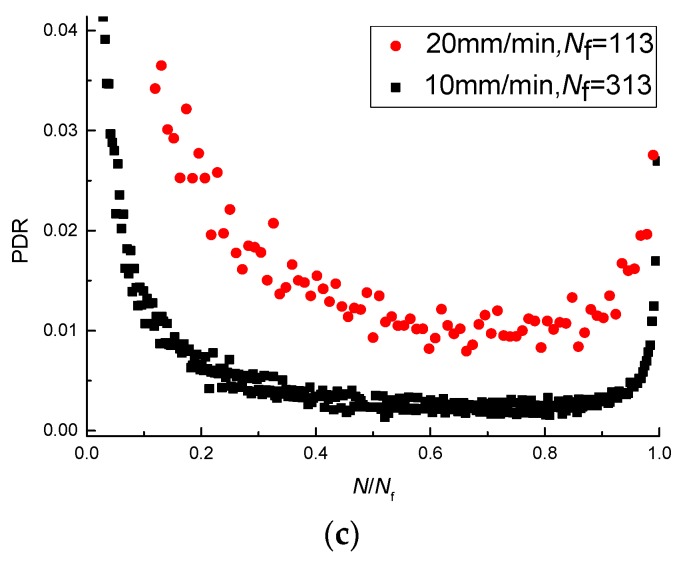
PDRs under different test conditions: (**a**) PDRs at different stress-strength ratios; (**b**) PDRs at different temperatures; (**c**) PDRs at different loading rates.

The PDRs at different loading cycles were calculated using Equation (4). To better analyze the PDR under different experimental factors, the number of loading cycles is converted into dimensionless quantities. As shown in Figure 10, at first, the PDR decreases with the increase of the number of loading cycles, then the ratio increases, and the permanent-deformation-ratio plot can be divided into three stages. The trends of the PDRs (vs. the number of loading cycles) under different test conditions are the same. The PDR decreases with the increase of the stress-strength ratio and the loading rate and increases with the decrease of the temperature, so there is a negative correlation between fatigue life and the PDR.

### 5.2. Analysis of Damage Evolution

Under certain conditions, the PD of the viscoelastic deformation part of each loading cycle is a fixed value, and the value of the PDR is only related to the damage deformation. Therefore, the damage factor can be expressed based on the PDR as

(5) $$D=\frac{\sum_{k=1}^{N}{\mathrm{PDR}}_{k}}{\sum_{k=1}^{N_{f}}{\mathrm{PDR}}_{k}}$$

where *D* is the damage factor of the loading cycle *N* and PDR*_k_* is the PDR at the loading cycle *k*.

**Figure 11:**
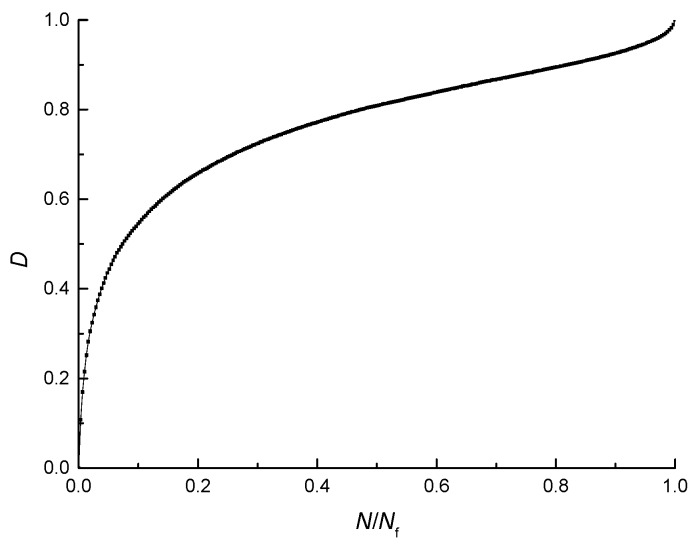
D-*N*/*N*_f_ curve.

**Figure 12:**
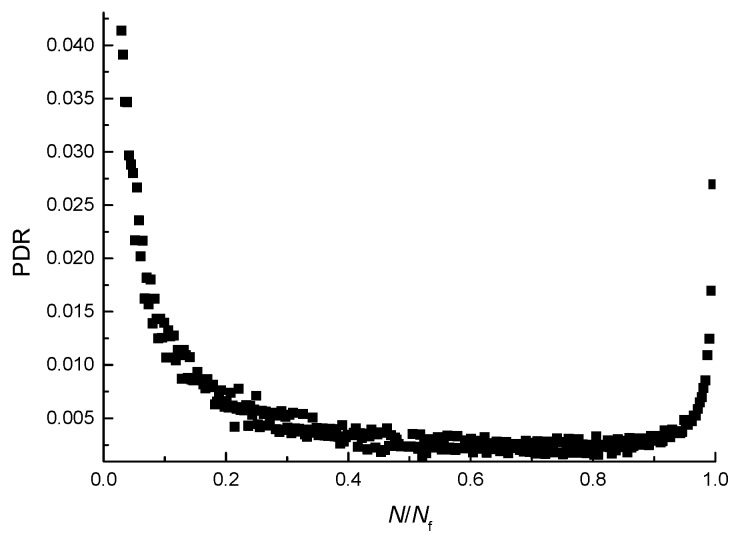
PDR-*N*/*N*_f_ scatter plot.

In order to study the damage evolution process of the asphalt mixture from the perspective of deformation, the damage evolution curve (the D-*N*/*N*_f_ curve) and the PDR evolution scatter plots (PDR-*N*/*N*_f_ scatter plot) for the fatigue test at the temperature of 25 °C, at the stress-strength ratio of 0.4, and at the loading rate of 10 mm/min are shown in Figure 11 and Figure 12. To better analyze the damage evolution process, the number of loading cycles is converted into dimensionless quantities.

As shown in Figure 11, the damage increases nonlinearly during the fatigue test, and the process can be divided into three stages. In the first stage, the damage lasts a short time but increases rapidly, and at the end of this stage, it reaches a large value. In the second stage, the damage lasts a long time and increases stably. In the third stage, the damage increases sharply and the beam specimen fractures in the end [42].

As shown in Figure 12, at first, the PDR decreases with the increase of the number of loading cycles, then the ratio increases, and the permanent-deformation-ratio plot can be divided into three stages. The first stage lasts a short time and the PDR decreases rapidly, but the speed of decreasing gradually slows down. At the end of the first stage, the PDR drops to a small value. The second stage lasts a long time and the PDR decreases slowly. The third stage lasts a short time, but the PDR increases sharply and the growth rate of the PDR increases gradually. The rapid decline in the first stage of the PDR shows that a considerable portion of the PD contributes to material damage. After the material damages to a certain extent, the PDR moves into the second stage of the low stable value, indicating that the PD mainly contributes to the viscoelastic deformation in the material. The PD of this part tends to be stable, which is far greater than what the PD contributed to material damage, which makes up a large proportion of the total deformation. In the third stage, the PD reaches the failure threshold and the specimen breaks quickly because of the deformation in the material accumulated in the first and second stage.

As shown in Figure 11 and Figure 12, the three stages of the PDR and the three stages of the damage correspond to each other, which indicates that there exists a connection between the damage evolution and the value of PDR. It can be seen from the growth rate of the damage and the value of PDR that the damage of the asphalt mixture increases rapidly in the first and third stage and the growth rates of damage in the first and third stage are greater than that in the second stage. In the same way, the values of the PDR in the first and third stage are greater than that in the second stage. Therefore, the PDR is an energy parameter reflecting the speed of damage evolution of the asphalt mixture, and the speed of damage evolution increases with the PDR of the asphalt mixture.

The faster the damage evolution speed of the asphalt mixture under cyclic loading is, the earlier the damage is close to the threshold of failure and the shorter the corresponding fatigue life is. Therefore, the fatigue life decreases with the increase of the PDR.

### 5.3. Establishment of Fatigue Equation Based on Plateau Value (PV) of PDR

The damage evolution analysis in Section 5.2 showed that fatigue life decreases with the increase of the PDR. At first, the PDR decreases with the increase in the number of loading cycles, then the ratio increases, and the process can be divided into three stages. Although the PDR in the first and third stages is greater than that in the second stage, the second stage accounts for the major portion of the entire PD in the loading process. Therefore, the PDR in the second stage reflects the overall PDR of the three stages. In this paper, the average PDR in the second stage is taken as the plateau value (PV), and the PDR of the three stages is represented by the PV.

**Figure 13:**
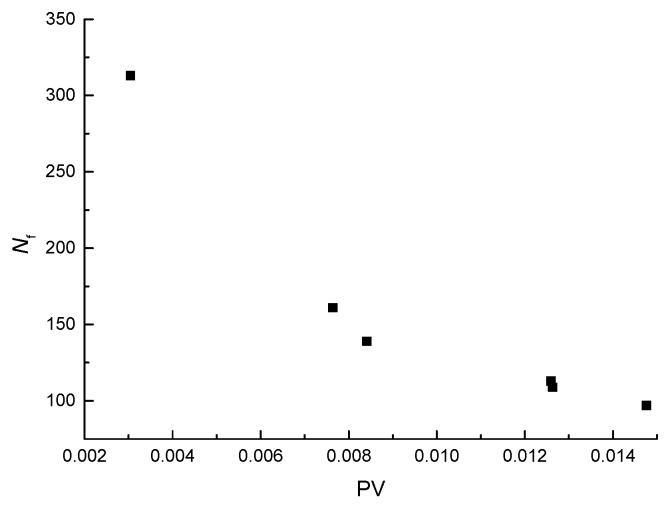
Scatter plots of *N*_f_-PV.

The results of the fatigue life (*N*_f_) and the PV of the three-point bending fatigue tests, as listed in Table 6, indicate that fatigue life decreases with the increase of the plateau value (PV). The *N*_f_-PV scatter plot is shown in Figure 13, which indicates that fatigue life can be expressed in a power function with the increase of PV. The power function, simple and easy to use, is established as

*N*_f_ = *A*(PV)*^B^* (6)

where *N*_f_ is the fatigue life and *A* and *B* are fitting parameters. The fitting results are listed in Table 7. The correlation coefficient is greater than 0.98, so the observed data are well represented by the equation.

According to the fatigue equation for the asphalt mixture, the predicted fatigue lives of the asphalt mixture under different test conditions were calculated and compared with the test fatigue lives, as listed in Table 8. The average relative error of the fatigue life predicted by the fatigue equation to the test fatigue life is 11.03%. Therefore, the fatigue equation based on the plateau value of the PDR can well predict the fatigue life of the asphalt mixture under cyclic loading.

## 6. Conclusions

Laboratory investigations of the change law of deformation of an asphalt mixture under cyclic loading and prediction of the fatigue life based on the PV of PDR were carried out by three-point bending fatigue tests. With the study above, the following conclusions can be drawn:

(1) The recovery ratio of the elastic deformation of the asphalt mixture under cyclic loading decreases with the increase of the number of loading cycles, and the elastic recovery ability of the asphalt mixture under cyclic loading decreases gradually. The proposed RRED mathematical model in this paper can well describe the change law of the RRED.
(2) The process of damage evolution of the asphalt mixture is accompanied by the accumulation of PD, and the accumulation of PD will further the evolution of damage. One part of the PD causes the damage of the asphalt mixture under cyclic loading, and the other part causes the viscoelastic deformation of the asphalt mixture. The proposed PD mathematical model can well describe the change law of RRED.
(3) The RRED or the PD cannot well predict the fatigue life of the asphalt mixture because the RRED and the PD of the asphalt mixture do not change monotonously with the fatigue life.
(4) The definition of PDR is proposed in this paper. The influence of experimental factors on the PDR indicates that the PDR increases with the stress-strength ratio and the loading rate, and increases with the decrease of the temperature, so there exists a negative correlation between fatigue life and the PDR.
(5) The PDR reflects the proportion of the PD that produces the viscoelastic deformation to the total PD of each loading cycle, and the value of the PDR is only related to the damage deformation. The damage evolution based on the PDR indicates that the faster the damage evolution speed is, the earlier the damage is close to the threshold of failure, and the fatigue life decreases with the increase of PDR.
(6) The average PDR in the second stage is taken as the PV, and the overall status of the PDR of the three stages and the resistance to fatigue damage are represented by the PV. The fatigue life decreases in power law with the increase of the PV. The average relative error of the fatigue life predicted by the fatigue equation to the test fatigue life is 5.77%. The fatigue equation based on the PV can well predict the fatigue life of the asphalt mixture.

## Figures and Tables

**Table 1:** Properties of asphalt rubber.

Material Property	Used Standard	Value
Softening point	T0606—2000	57 (°C)
Viscosity	T0625—2000	1.5–4.0 (Pa·s)
Elastic recovery	T0662—2000	30 (%)

**Table 2:** Aggregate gradation.

Sieve Size (mm)	16.0	13.2	9.5	4.75	2.36	1.18	0.6	0.3	0.15	0.075
Passing percentage	100.0	91.1	80.2	54.0	33.2	22.5	16.0	12.1	8.7	5.5

**Table 3:** Fatigue lives.

SSR-Temperature-Loading Rate	Value
0.6–25 °C-10 mm/min	313
0.7–25 °C-10 mm/min	161
0.8–25 °C-10 mm/min	139
0.6–15 °C-10 mm/min	109
0.6–5 °C-10 mm/min	97
0.6–25 °C-20 mm/min	113

**Table 4:** Fitting results of RRED mathematical model.

Fitting Parameters	*a*	*b*	*R*^2^
Value	0.25937	−0.37801	0.99456

**Table 5:** Fitting results of PD mathematical model.

Parameter	*c*	*d*	*R*^2^
Parameter value	0.85777	0.56389	0.98372

**Table 6:** Results of *N*_f_ and PV.

Test Fatigue Life (*N*_f_)	PV
313	0.00303819
161	0.007641095
139	0.008407475
113	0.012586557
109	0.014762424
97	0.012631972

**Table 7:** Fitting parameters of fatigue equation.

Parameter	*A*	*B*	*R*^2^
Parameter value	4.24479	−0.74172	0.98653

**Table 8:** Contrast results of fatigue life.

SSR-Temperature-Loading Rate	Test Fatigue Life	Predicted Fatigue Life	Relative Error
0.6–25 °C-10mm/min	313	312	0.11%
0.7–25 °C-10mm/min	161	158	2.02%
0.8–25 °C-10mm/min	139	147	5.72%
0.6–25 °C-20mm/min	113	109	3.59%
0.6–15 °C-10mm/min	109	97	11.20%
0.6–5 °C-10mm/min	97	109	12.00%
